# The Utility of Radiomorphometric Mandibular Indices on Cone Beam Computer Tomography in the Assessment of Bone Mass in Postmenopausal Women: A Cross-Sectional Study

**DOI:** 10.3390/jpm14080843

**Published:** 2024-08-09

**Authors:** Ioana Ruxandra Poiană, Ramona Dobre, Silviu-Mirel Pițuru, Alexandru Bucur

**Affiliations:** 1 Faculty of Medicine, “Carol Davila” University of Medicine and Pharmacy, 050474 Bucharest, Romania; ioana.poiana@drd.umfcd.ro (I.R.P.); silviu.pituru@umfcd.ro (S.-M.P.); alexandru.bucur@umfcd.ro (A.B.); 2 Department of Endocrinology, National Institute of Endocrinology C. I. Parhon, 011853 Bucharest, Romania

**Keywords:** osteoporosis, menopause, cone beam computed tomography, radiomorphometric mandibular indices

## Abstract

Background: The present study examined the potential use of computed tomography radiomorphometric mandibular indices on cone beam CT (CBCT) for the assessment of bone density in postmenopausal women with low bone mass. Methods: We included 104 postmenopausal women who received dual-energy X-ray absorptiometry (DXA) using a DXA scanner and mental foramen (MF) region CBCT using a NewTom VGi EVO Cone Beam 3D system. We assessed the relationships between the following DXA parameters: lumbar, femoral neck, and total hip T-scores, bone mineral density (BMD), lumbar trabecular bone score (TBS), and mandibular inferior cortical bone thickness at 4 sites. The cross-sectional images were obtained, as follows: anterior (A)—10 mm anterior from the MF; molar (M)—10 mm posterior from the MF; posterior (P)—25 mm posterior from the MF; symphysis (S)—equidistant from the centers of the right and left MF. Results: We found that A and M indices showed statistically significant moderate positive correlations with lumbar spine, femoral neck, and total hip BMD, as well as TBS. The P index demonstrated moderate positive correlations with these measurements, while the S index did not show significant correlations with BMD or TBS in postmenopausal women. Conclusions: These findings support the potential usefulness of CBCT-derived radiomorphometric mandibular indices for non-invasive bone health assessment in clinical practice.

## 1. Introduction

Osteoporosis is a chronic disorder, being a major public health issue with an important impact on quality and quantity of life [1,2]. It is a preventable and treatable disease, yet only a small proportion of people with increased risk for fracture are indeed evaluated and treated [3,4]. It diminishes the mineral density and alters the bone microarchitecture, with increased marrow space, the result being a fragile bone prone to fracture with low or no traumatic mechanism [5,6]. The gold standard for diagnosis of low bone mass density (BMD) is dual energy X-ray absorptiometry (DXA).

Bone density is the main factor influencing bone strength [7]. Osteoporosis, particularly in postmenopausal women, significantly influences the success of dental implants due to the associated decrease in bone density and structural integrity of the jawbone that can compromise the stability and integration of dental implants [8], adequate bone density being crucial for the osseointegration process, where the implant fuses with the bone. In osteoporotic patients, the compromised bone quality can lead to reduced primary stability, increasing the risk of implant failure or delayed stability [2,8,9,10]. Although osteoporosis is viewed as a relative contraindication for implant therapy due to delayed osteointegration and elevated crestal bone loss [6], some studies showed no important effect of low bone mass around dental implants [11]. Given these challenges, careful planning and management are essential when considering dental implants for postmenopausal women with osteoporosis, most of these cases requiring a multidisciplinary approach to ensure successful dental implant outcomes [12,13].

Cone beam computed tomography (CBCT) is used for the preoperative assessment of implant sites [8,14], with reduced radiation dose and low costs. This tool offers spatial resolution and gray density and also can be used to predict the BMD [15]. In consequence, different radiomorphometric indices were proposed for identifying low bone mass on CBCT images, the most commonly used in the literature being the mandibular cortex index, mandibular cortical width, and panoramic mandibular index [16,17], which appear to be the most useful for the initial detection of reduced BMD in patients. Compared to panoramic radiographs, CBCT allows 3D examination of anatomic structures with more detailed and high-quality images [12,18].

The most commonly used measurements are performed on the mental foramen (MF) region [19,20], knowing that it is not influenced by chewing muscles [21], and also because it has a fixed position. Gungor et al. [22] emphasized that osteoporotic bone changes could be accurately assessed using CBCT images. Also, there are several studies that found significant correlations between CBCT radiomorphometric indices and BMD based on DXA evaluation in patients with osteoporosis [23,24]. The findings of Barra et al. [21] demonstrated that the molar and posterior indices can be useful mandibular CBCT tools in the evaluation of bone density and therefore in the diagnosis of osteoporosis in postmenopausal women. The CBCT-derived indices evaluated are very similar to the MCW used in panoramic radiographs, but in different locations in the mandible [19,25,26].

Our purpose was to evaluate the correlations between radiomorphometric mandibular indices and bone mass density in postmenopausal women, regarding both quantity and quality of the bone assessed by DXA and trabecular bone score (TBS).

## 2. Materials and Methods

This is a cross-sectional study, performed on 104 postmenopausal women, with normal BMD, osteopenia, or osteoporosis, with or without antiresorptive/anabolic treatment.

The inclusion criteria were as follows: female sex, menopausal status, DXA with TBS evaluation, biochemical evaluation, CBCT evaluation. Exclusion criteria were as follows: presence of systemic diseases affecting bone metabolism (neoplasia, osteomalacia, history of rickets, and endocrine disorders, like hyperthyroidism, hyperparathyroidism, Cushing’s syndrome, or acromegaly), severe renal failure, liver failure, malabsorption disorders, rheumatologic diseases (rheumatoid arthritis, ankylosing spondylitis), history of oophorectomy, usage of medications interfering with bone density (glucocorticoids, aromatase inhibitors, selective serotonin reuptake inhibitors, medroxyprogesterone acetate, antiepileptic drugs, unfractionated heparin).

The patients included in the study were future or previous candidates for dental implant and they were required to do a CBCT as part of the preimplantation protocol commonly used in our country (most of the institutions are private practices with personalized protocols, but CBCT evaluation is very commonly used in most of the dental clinics). The patients were evaluated in close collaboration with an important private provider of dental imagistics with expertise in CBCT imaging, and respectively, the National Institute of Endocrinology, a public hospital that attends hundreds of patients with metabolic bone pathologies every year.

Written informed consent was obtained from patients before the study. The study was approved by the Ethics Committee of “C. I. Parhon” National Institute of Endocrinology, Bucharest, Romania (No. 4/08.04.2021).

### 2.1. CBCT Measurements

The CBCT images were obtained using NewTom VGi EVO Cone Beam 3D Imaging, version 16.2.2 ß2 (CEFLA s.c.—Via Selice Provinciale 23/a IMOLA, Imola, Italy), at 110 kV, 7.5 mA, 3.5 s, pixel size 0.2 mm. The images were reconstructed using NewTom NNT, version 4.0 (ISDP©10003:2020 compliant in accordance with [27] certificate number 2019003109-2) with Viewer software, version 16.2.2 ß2. Most of the evaluations had a maximum DAP of 722.69 mGy * cm^2^ and CTDI of 3.6 mGy.

**Figure 1:**
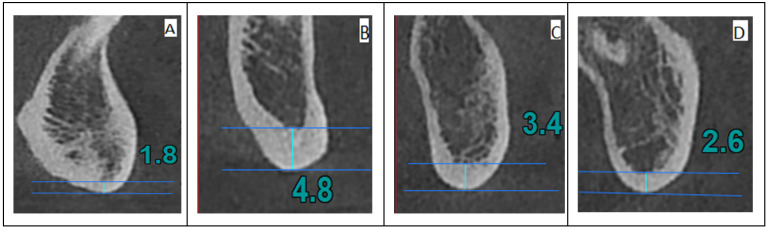
Index measurements in cross-sectional images: (**A**) (S index, symphysis)—the thickness of the mandibular inferior cortex equidistant from the centers of the right and left mental foramina; (**B**) (A index, anterior)—the thickness of the mandibular inferior cortex 10 mm anterior to the mental foramina; (**C**) (M index, molar)—the thickness of the mandibular inferior cortex 10 mm posterior to the mental foramina; (**D**) (P index, posterior)—the thickness of the mandibular inferior cortex 25 mm posterior to the mental foramina.

We analyzed cross-sectional CBCT images in 4 sites (see Figure 1), identified according to the mental foramen (the standard region for BMD evaluation), as follows:

- Anterior index (A)—the thickness of the inferior mandibular cortex 10 mm anterior from the MF;
- Molar index (M)—the thickness of the inferior mandibular cortex 10 mm posterior from the MF;
- Posterior index (P)—the thickness of the inferior mandibular cortex 25 mm posterior from the MF;
- Symphysis index (S)—the thickness of the inferior mandibular cortex equidistant from the centers of the right and left MF.

### 2.2. Bone Mineral Density Measurements

BMD was measured at the lumbar spine (LS), femoral neck (FN), and total hip by DXA (General Electric Prodigy Lunar, Bedford, UK) using an enCore Software 10,50,086. BMD was expressed in grams per square centimeter (g/cm^2^), and by comparing the BMD with the peak bone mass of a young adult, a T-score was obtained, expressed in standard deviations (SD), and a Z-score was obtained for age-matched SD [15]. All measurements were performed according to the International Society for Clinical Osteodensitometry [28].

TBS values were obtained by analyzing the L1–L4 vertebrae DXA images with iNsight Software version 2.2.0.0 (Medimaps Group SA Headquarters, Plan-les-Ouates, Switzerland).

All patients were scanned on the same DXA machine by two different operators, thus allowing a user bias.

According to the 2020 American Association of Clinical Endocrinologists (AACE) guidelines, the diagnosis of osteoporosis in postmenopausal women is based on the following criteria [3]:

- T-score −2.5 or below in the lumbar spine, femoral neck, total proximal femur, or 1/3 radius;
- Low-trauma spine or hip fracture (regardless of bone mineral density);
- T-score between −1.0 and −2.5 and a fragility fracture of the proximal humerus, pelvis, or distal forearm;
- T-score between −1.0 and −2.5 and high fracture risk assessment tool (FRAX^®^ University of Sheffield, UK) fracture probability based on country-specific thresholds.

### 2.3. Statistical Analysis

We statistically analyzed the patients based on the value of the BMD (lumber spine, femoral neck, total hip) and T-score (lumber spine, femoral neck, total hip), respectively, and TBS, as continuous values, regardless of the osteoporosis diagnosis at the time of CBCT evaluation.

We also used binary logistic analysis to divide the patients based on the osteoporosis diagnosis (according to AACE/ACE criteria) [2]. We used parametric tests, regression analysis, the *t*-test, the Pearson’s correlation coefficient, and Spearman’s rho, using IBM SPSS Statistics, version 25 (SPSS Inc., Chicago, IL, USA) for Mac OS.

## 3. Results

The present study evaluated the relationship between computed tomography radiomorphometric mandibular indices obtained from cone beam CT (CBCT) and BMD measurements from DXA in postmenopausal women. The patent distribution is listed in Table 1 below.

Lumbar T-score in osteoporotic patients was −3.12 SD, compared to the second group with −1.3 SD.

Table 2 lists the mean values (in millimeters) of the four analyzed radimorphometric indices in the BMD groups.

Higher values of the indices are observed in patients with normal bone quality compared to patients with intermediate or low quality (TBS lower than 1.310).

A statistically significant moderate positive correlation was found between anterior and molar indices (A and M) and lumbar T-score (*p* < 0.0001, r = 0.387, respectively *p* < 0.0001, r = 0.429), the posterior index (P) having a positive correlation with the lumbar T-score (*p* = 0.004, r = 0.285). No significant correlation was found between lumbar T-score and symphysis index (S) in postmenopausal women (*p* = 0.675, r = 0.043). The correlation results were similar regarding the studied indices and femoral neck and total hip T-scores (see Table 3).

In this study, both Spearman and Pearson correlation coefficients were used to analyze the relationship between mandibular CBCT indices and BMD and TBS measurements (Table 4). The anterior index shows statistically significant moderate Pearson correlations with all three BMD measurements and TBS, indicating that as the anterior index increases, BMD and TBS also tend to increase linearly. The molar index has even stronger positive Pearson correlations with BMD and TBS than the anterior index. The posterior index shows moderate positive Pearson correlations, suggesting it is a less strong but still significant predictor of BMD and TBS. The symphysis index shows no significant Pearson correlation with any BMD measurements or TBS, indicating that it does not linearly relate to bone density or quality, with some exceptions using Spearman’s at the hip level and with TBS.

Logistic regression analysis was used to evaluate the predictive value of computed tomography radiomorphometric mandibular indices from CBCT for osteoporosis and bone quality (see Table 5). The anterior and molar indices are predictors for osteoporosis, with better predictive power than the posterior index, results being very similar between anterior and molar indices. The posterior index is also a significant predictor, though slightly less strong than the anterior and molar indices. The symphysis index does not significantly predict osteoporosis or low bone quality.

With regards to bone quality evaluated by TBS, the predictive values are significantly higher for A, M, and P indices, *p* < 0.0001.

Using the logistic regression analysis, the symphysis (S) index had an odds ratio of 1.26 (*p* = 0.384), while the anterior (A), molar (M), and posterior (P) indices had significant odds ratios with *p*-values below 0.001 in predicting low bone quality (TBS ≤ 1.23) and osteoporosis defined by lumbar and femoral neck T-scores. There were also significant predictive values for the anterior, molar, and posterior indices, but not for the symphysis index. The regression values (R^2^) for these indices were highest for the molar index, followed by the anterior and posterior indices.

## 4. Discussion

The present study explored the potential role of computed tomography radiomorphometric mandibular indices measured with CBCT in assessing bone quality and quantity in postmenopausal women with low bone mass. We found significant correlations between mandibular indices obtained from CBCT and bone mineral density (BMD) measurements from DXA and also bone quality assessed with TBS. This is the first study to evaluate the possible correlation of CBCT-derived indices and bone quality. Specifically, the anterior (A), molar (M), and posterior (P) indices showed strong associations with lumbar spine and femoral neck T-scores.

Menopause, characterized by an important decline in estrogen levels, plays a crucial role in maintaining bone density secondary to its effect on regulating bone remodeling by balancing bone formation and resorption [29]. This effect leads to increased bone resorption and decreased bone formation, resulting in overall bone loss and increased risk of osteoporosis. This systemic effect was also evident in the jaw, making it susceptible to similar alterations seen in other skeletal sites [30].

Studies have shown that postmenopausal women exhibit decreased bone mineral density (BMD) in the mandible, which can compromise the structural integrity needed for dental implant success [19]. Taguchi et al. [31,32] reinforced these findings by showing that dental radiographs and DXA scans could effectively detect osteoporotic changes in the jaw, providing crucial diagnostic tools for assessing implant feasibility.

Reduced bone density in osteoporotic patients can lead to decreased primary stability of the implants, as discussed by Taguchi [32,33], making them more prone to micromovements and failure during the healing phase. Contrastingly, Wang [18] noted that while the link between periodontal disease and osteoporosis was evident, the severity of bone loss in the jaw could vary widely among individuals, indicating the need for personalized treatment plans. These findings collectively emphasize the need for comprehensive evaluation and tailored management strategies for postmenopausal women considering dental implants, addressing the specific challenges posed by menopause-induced bone alterations in relation to both quantity and quality [2,34].

The first study that evaluated BMD using mandibular measurements in postmenopausal women was performed by Koh et al. [23] and used superior and inferior cortical indices (CTI(S) and CTI(I)), with significant differences between normal and osteoporotic groups (*p* < 0.05). Since then, other authors have tried to confirm these results or find other CBCT-derived indices to assess the bone mass in this particular population at risk [8].

In our study, we evaluated several regions of the mandible (10 mm anterior, 10 mm posterior, 25 mm posterior from the MF) as new mandibular indices in order to validate them as reliable substitutes in the assessment of low BMD in postmenopausal women. The most commonly used quantitative indices for the determination of low BMD in the MF region in the literature are the panoramic mandibular index (PMI) and the mental index (MI), also known as the mandibular cortical width (MCW) [8,35,36]. These new indices in CBCT proposed by Barra et al. [21] are very similar to the MCW used in panoramic radiographs but in different locations in the mandible. Similar to the radiomorphometric indices in panoramic radiographs, the CBCT indices had lower values in patients with low BMD compared with healthy individuals [24].

The anterior, molar, and posterior indices from CBCT images show significant correlations with lumbar spine T-scores. These indices can effectively reflect the BMD of the lumbar spine, with the anterior and molar indices being the most reliable indicators. The symphysis index, however, does not show a significant correlation, suggesting it may not be as useful for assessing lumbar BMD. Similar to the lumbar T-score, the anterior, molar, and posterior indices are significantly correlated with the femoral neck and total hip T-scores, not evaluated in other studies, with the molar index showing the strongest correlation. Our findings are in concordance with the results of Barra et al. [21] regarding the M index and also for the P index. These indices exhibited significant differences among patients with normal BMD and those with osteopenia and/or osteoporosis; for the A and S indices, they did not find significant differences between the normal group and the osteoporosis and/or osteopenia groups. Their results had high sensitivity, although low specificity (37.5%) [21].

While DXA remains the gold standard for diagnosing osteoporosis, this study found that CBCT could complement DXA by providing additional insights into bone quality. The CBCT indices not only correlated with BMD but also with TBS, which is an indicator of bone microarchitecture, an important determinant of bone strength and fracture risk independent of BMD. The molar and anterior indices showed strong correlation with TBS, suggesting that the cortical thickness measured at the anterior and molar regions of the mandible are good indicators of trabecular bone microarchitecture quality. A higher anterior index corresponds to a higher TBS, indicating better trabecular structure and lower fracture risk. The posterior index shows a positive correlation with TBS, although the correlation is weaker compared to the anterior and molar indices. This indicates that while the posterior mandibular cortical thickness can reflect trabecular bone quality, it may not be as strong a predictor as the anterior and molar indices. The symphysis index shows no significant correlation with TBS, so it may not be useful for assessing trabecular bone health. This underlines the possible utility of CBCT in a clinical setting, especially for patients who may not have access to DXA or TBS evaluation.

Patients with a lumbar T-score > −2.5 SD had significantly higher mean CBCT values across all indices compared to those with a lumbar T-score ≤ −2.5 SD. Similar trends were observed for femoral neck T-scores and total hip T-scores. The patients with normal or mildly reduced BMD (T-score > −2.5 SD) have thicker mandibular cortices in CBCT images compared to those with osteoporosis (T-score ≤ −2.5 SD). This indicates that CBCT can distinguish between different degrees of bone density.

The strengths of our study reside in the higher number of patients compared to other studies that evaluated CBCT-derived indices [8] and also in the more complex assessment of osteoporosis, not only as T-score at the lumbar, or, in fewer studies, lumbar plus femoral neck, but also total hip and patients already diagnosed with osteoporosis based on AACE criteria, including fracture assessment as an important indicator of poor bone mass. Also, this is the first study to assess the correlations of these indices with bone quality assessed by TBS.

We acknowledge certain limitations of the study, such as the relatively small sample size and its cross-sectional type. Future research could expand on these findings by including larger, more diverse populations and exploring longitudinal changes in bone density. The symphysis index showed no significant correlations, suggesting that not al CBCT indices are equally useful. The deficit of research on radiomorphometric indices of the maxilla might be attributed to the unique challenges of the analyzed images, which lack distinct reference points necessary for consistent measurements. Furthermore, the jaw consists of relatively thin bones, and the abundance of spongy bone complicates imaging. Despite these challenges, these characteristics make the jaw a reliable indicator of changes in bone mineral density. The idea of combining the analysis of indicators can increase the specificity value, meaning a better ability to exclude healthy individuals from further diagnosis of low bone mass.

Additionally, investigating the cost-effectiveness and accessibility of CBCT in various clinical settings is important. Our findings can enhance osteoporosis screening and management in clinical settings, emphasizing the importance of existing dental imaging technologies.

## 5. Conclusions

Our study demonstrated that the evaluated CBCT mandibular indices in the anterior and posterior regions from the MF can identify low bone mass in patients with osteoporosis and can also reliably assess bone quality. When evaluating CBCT images, radiologists and dental care providers should also evaluate the changes in bone density in the mandibular area and correlate them with the risk of implant failure and peri-implant bone stability.

## Figures and Tables

**Table 1:** Patient characteristics.

Parameter	T-Score ≤ −2.5 *	T-Score ≥ −2.5 *
Number	45	59
Age at menopause (years)	47.18 ± 4.49	47.43 ± 5.73
Femoral neck T-score (SD)	−2.06 ± 0.66	−1.15 ± 0.85
Total hip T-score (SD)	−1.67 ± 0.78	−0.6 ± 1.04
Lumbar T-score (L1–L4) (SD)	−3.12 ± 0.69	−1.3 ± 0.92
Femoral neck BMD (g/cm^2^)	0.727 ± 0.082	0.852 ± 0.11
Total hip BMD (L1–L4) (g/cm^2^)	0.713 ± 0.366	0.941 ± 0.132
Lumbar BMD (g/cm^2^)	0.805 ± 0.088	1.018 ± 0.012
TBS score (g/cm^2^)	1.207 ± 0.181	1.313 ± 0.101

* The values are expressed as mean ± standard deviation (SD), not based on osteoporosis AACE criteria.

**Table 2:** Mean values of the computed tomography parameters on cone beam computed tomography (CBCT) images.

CBCT Parameter	Lumbar T-Score > −2.5 SD (n = 62)	Lumbar T-Score ≤ −2.5 SD (n = 42)	Low Bone Quality (TBS ≤ 1.23, n = 21)	Intermediate Bone Quality (TBS > 1.23 and <1.31, n = 32)	Normal Bone Quality (TBS ≥ 1.31, n = 33)	Femoral Neck T-Score > −2.5 DS (n = 85)	Femoral Neck T-Score ≤ −2.5 DS (n = 14)	*p* Value
S	2.03 ± 0.6	1.73 ± 0.57	1.85 ± 0.63	1.75 ± 0.49	2.0 ± 0.63	1.9 ± 0.59	1.75 ± 0.55	<0.001
A	2.93 ± 0.91	2.30 ± 0.72	2.22 ± 0.67	2.41 ± 0.85	2.9 ± 0.78	2.6 ± 0.78	1.65 ± 0.47	<0.001
M	2.72 ± 0.74	1.9 ± 0.6	2.04 ± 0.65	2.05 ± 0.82	2.6 ± 0.75	2.3 ± 0.76	1.57 ± 0.47	<0.001
P	2.6 ± 0.66	2.04 ± 0.69	2.17 ± 0.69	2.26 ± 0.71	2.5 ± 0.72	2.4 ± 0.70	1.71 ± 0.54	<0.001

SD, standard deviation; CBCT, cone beam computer tomography; anterior (A)—10 mm anterior from the mental foramina, MF; molar (M)—10 mm posterior from the MF; posterior (P)—25 mm posterior from the MF; symphysis (S) equidistant from the centers of the right and left MF.

**Table 3:** Correlations between CBCT parameters and bone quantity and quality parameters.

Parameters for Correlations ^†^	S	A	M	P
Lumbar T-score *	0.043, *p* = 0.675	0.387, *p* < 0.0001	0.429, *p* < 0.0001	0.285, *p* = 0.004
Femoral neck T-score *	0.079, *p* = 0.44	0.468, *p* < 0.0001	0.515, *p* < 0.0001	0.363, *p* < 0.0001
Total hip T-score *	0.143, *p* = 0.16	0.441, *p* < 0.0001	0.492, *p* < 0.0001	0.410, *p* < 0.0001
TBS score ^#^	0.125, *p* = 0.25	0.48, *p* < 0.0001	0.420, *p* < 0.0001	0.291, *p* = 0.006
TBS quality assessment ***	0.153, *p* = 0.156	0.480, *p* < 0.0001	0.465, *p* < 0.0001	0.341, *p* < 0.001
Lumbar BMD **	−0.004, *p* = 0.968	0.368, *p* < 0.0001	0.369, *p* < 0.0001	0.240, *p* = 0.017
Femoral neck BMD **	0.11, *p* = 0.28	0.478, *p* < 0.0001	0.526, *p* < 0.0001	0.387, *p* < 0.0001
Total hip BMD **	0.162, *p* = 0.11	0.428, *p* < 0.0001	0.52, *p* < 0.0001	0.389, *p* < 0.0001
Osteoporosis defined as lumbar T-score ≤ −2.5 SD	0.028, *p* = 0.87	0.326, *p* = 0.052	0.281, *p* = 0.098	0.002, *p* = 0.99

^†^ Significant at the 0.05 and 0.01 level *t*-test (2-tailed). * Expressed as standard deviations; ** bone mass density, expressed as g/cm^2^; *** trabecular bone score, expressed as low if TBS ≤ 1.23 g/cm^2^, intermediate if TBS > 1.23 and <1.31 g/cm^2^, and normal if TBS > 1.31 g/cm^2^; ^#^ as continuous value. TBS, trabecular bone score, expressed as g/cm^2^; SD, standard deviation; CBCT, cone beam computer tomography; anterior (A)—10 mm anterior from the mental foramina, MF; molar (M)—10 mm posterior from the MF; posterior (P)—25 mm posterior from the MF; symphysis (S) equidistant from the centers of the right and left MF.

**Table 4:** CBCT parameters and bone quantity and quality parameters by Pearson’s correlation coefficient and Spearman’s rho.

Pearson’s Correlation ^†^	S	A	M	P
Lumbar T-score *	0.043, *p* = 0.67	0.387, *p* < 0.0001	0.429, *p* < 0.0001	0.285, *p* < 0.0001
Femoral neck T-score *	0.079, *p* = 0.43	0.468, *p* < 0.0001	0.515, *p* < 0.0001	0.363, *p* < 0.0001
Total hip T-score *	0.143, *p* = 0.15	0.441, *p* < 0.0001	0.492, *p* < 0.0001	0.410, *p* < 0.0001
TBS score ^#^	0.125, *p* = 0.24	0.480, *p* < 0.0001	0.420, *p* < 0.0001	0.291, *p* = 0.006
TBS quality assessment ***	0.153, *p* = 0.15	0.480, *p* < 0.0001	0.465, *p* < 0.0001	0.341, *p* < 0.001
Lumbar BMD **	−0.004, *p* = 0.968	0.368, *p* < 0.0001	0.369, *p* < 0.0001	0.240, *p* = 0.017
Femoral neck BMD **	0.11, *p* = 0.28	0.478, *p* < 0.0001	0.526, *p* < 0.0001	0.387, *p* < 0.0001
Total hip BMD **	0.162, *p* = 0.11	0.428, *p* < 0.0001	0.520, *p* < 0.0001	0.389, *p* < 0.0001
Osteoporosis defined as lumbar T-score ≤ −2.5 SD	0.028, *p* = 0.87	0.326, *p* = 0.052	0.281, *p* = 0.098	0.002, *p* = 0.99
Osteoporosis defined as femoral neck T-score ≤ −2.5 SD	−0.043, *p* = 0.81	0.29, *p* = 0.1	0.278, *p* = 0.11	0.11, *p* = 0.53
**Spearman’s rho**				
Lumbar T-score *	0.166, *p* = 0.1	0.379, *p* < 0.0001	0.437, *p* < 0.0001	0.281, *p* < 0.0001
Femoral neck T-score *	0.277, *p* = 0.005	0.458, *p* < 0.0001	0.507, *p* < 0.0001	0.331, *p* < 0.0001
Total hip T-score *	0.311, *p* = 0.002	0.411, *p* < 0.0001	0.477, *p* < 0.0001	0.317, *p* < 0.001
TBS score ^#^	0.230, *p* = 0.026	0.507, *p* < 0.0001	0.448, *p* < 0.0001	0.296, *p* < 0.0001
TBS quality assessment ***	0.239, *p* = 0.026	0.492, *p* < 0.0001	0.485, *p* < 0.0001	0.329, *p* < 0.0001
Lumbar BMD **	0.10, *p* = 0.32	0.373, *p* < 0.0001	0.373, *p* < 0.0001	0.217, *p* = 0.032
Femoral neck BMD **	0.315, *p* = 0.002	0.456, *p* < 0.0001	0.5, *p* < 0.0001	0.339, *p* < 0.001
Total hip BMD **	0.355, *p* < 0.0001	0.389, *p* < 0.0001	0.486, *p* < 0.0001	0.320, *p* < 0.001
Osteoporosis defined as lumbar T-score ≤ −2.5 SD	0.038, *p* = 0.826	0.353, *p* = 0.034	0.327, *p* = 0.052	0.003, *p* = 0.98
Osteoporosis defined as femoral neck T-score ≤ −2.5 SD	0.1, *p* = 0.58	0.285, *p* = 0.1	0.232, *p* = 0.194	−0.002, *p* = 0.99

^†^ Significant at the 0.05 and 0.01 level *t*-test (2-tailed). * Expressed as standard deviations; ** bone mass density, expressed as g/cm^2^; *** trabecular bone score, expressed as low if TBS ≤ 1.23 g/cm^2^, intermediate if TBS > 1.23 and <1.31 g/cm^2^, and normal if TBS > 1.31 g/cm^2^; ^#^ as continuous value. TBS, trabecular bone score, expressed as g/cm^2^; SD, standard deviation; CBCT, cone beam computer tomography; anterior (A)—10 mm anterior from the mental foramina, MF; molar (M)—10 mm posterior from the MF; posterior (P)—25 mm posterior from the MF; symphysis (S)—equidistant from the centers of the right and left MF.

**Table 5:** Predictions of bone quantity and bone quality using regression analysis.

**(a) Linear Regression**					
**Parameters**	**Variable**	**Regression Value**	**Constant of the Model**		**Model’s Sig.**
Lumbar T-score *	S	0.65, *p* = 0.675	−2.07, *p* < 0.0001		R^2^ = 0.002, *p* = 0.675
Femoral neck T-score *		0.855, *p* = 0.77	−1.63, *p* < 0.0001		R^2^ = 0.006, *p* = 0.43
Total hip T-score *		0.18, *p* = 0.14	−1.35, *p* < 0.0001		R^2^ = 0.021, *p* = 0.15
TBS score *^#^*		0.016, *p* = 0.24	1.254, *p* < 0.0001		R^2^ = 0.016, *p* = 0.24
Lumbar T-score *	A	0.634, *p* < 0.0001	−3.51, *p* < 0.0001		R^2^ = 0.15, *p* < 0.0001
Femoral neck T-score *		0.544, *p* < 0.0001	−2.82, *p* < 0.0001		R^2^ = 0.219, *p* < 0.0001
Total hip T-score *		0.6, *p* < 0.0001	−2.49, *p* < 0.0001		R^2^ = 0.194, *p* < 0.0001
TBS score *^#^*		0.006, *p* < 0.0001	1.128, *p* < 0.0001		R^2^ = 0.230, *p* < 0.0001
Lumbar T-score *	M	0.724, *p* < 0.0001	−3.58, *p* < 0.0001		R^2^ = −0.184, *p* < 0.0001
Femoral neck T-score *		0.625, *p* < 0.0001	−2.87, *p* < 0.0001		R^2^ = 0.265, *p* < 0.0001
Total hip T-score *		0.693, *p* < 0.0001	−2.56, *p* < 0.0001		R^2^ = 0.242, *p* < 0.0001
TBS score *^#^*		0.005, *p* < 0.0001	1.159, *p* < 0.0001		R^2^ = 0.177, *p* < 0.0001
Lumbar T-score *	P	0.52, *p* = 0.004	−3.14, *p* < 0.0001		R^2^ = 0.081, *p* < 0.0001
Femoral neck T-score *		0.474, *p* < 0.0001	−2.54, *p* < 0.0001		R^2^ = 0.132, *p* < 0.0001
Total hip T-score *		0.623, *p* < 0.0001	−2.42, *p* < 0.0001		R^2^ = 0.168, *p* < 0.0001
TBS score *^#^*		0.004, *p* = 0.006	1.185, *p* < 0.0001		R^2^ = 0.085, *p* = 0.006
**(b) Logistic Regression**					
**Parameters**	**Variable**	**Odds Ratio**		**Model’s Sig.**	
TBS quality assessment ***	S	1.26, 95% CI (0.74, 2.16)		*p* = 0.384	
Osteoporosis defined as lumbar T-score ≤ −2.5 SD *		0.9, 95% CI (0.53, 1.51)		*p* = 0.69	
Osteoporosis defined as femoral neck T-score ≤ −2.5 SD *		1.13, 95% CI (0.63, 2.06)		*p* = 0.68	
Osteoporosis based on AACE criteria **		0.79, 95% CI (0.48, 1.28)		*p* = 0.34	
TBS quality assessment ***	A	1.14, 95% CI (1.06, 1.21)		*p* < 0.0001	
Osteoporosis defined as lumbar T-score ≤ −2.5 SD *		0.92, 95% CI (0.87, 0.97)		*p* = 0.007	
Osteoporosis defined as femoral neck T-score ≤ −2.5 SD *		0.89, 95% CI (0.80, 0.97)		*p* = 0.014	
Osteoporosis based on AACE criteria **		0.9, 95% CI (0.85, 0.95)		*p* < 0.0001	
TBS quality assessment ***	M	1.14, 95% CI (1.06, 1.22)		*p* < 0.0001	
Osteoporosis defined as lumbar T-score ≤ −2.5 SD *		0.89, 95% CI (0.83, 0.95)		*p* = 0.001	
Osteoporosis defined as femoral neck T-score ≤ −2.5 SD *		0.83, 95% CI (0.75, 0.94)		*p* = 0.002	
Osteoporosis based on AACE criteria **		0.88, 95% CI (0.82, 0.94)		*p* < 0.0001	
TBS quality assessment ***	P	1.1, 95% CI (1.023, 0.174)		*p* = 0.009	
Osteoporosis defined as lumbar T-score ≤ −2.5 SD *		0.92, 95% CI (0.86, 0.98)		*p* = 0.008	
Osteoporosis defined as femoral neck T-score ≤ −2.5 SD *		0.86, 95% CI (0.76, 0.96)		*p* = 0.007	
Osteoporosis based on AACE criteria **		0.9, 95% CI (0.85, 0.97)		*p* = 0.002	

* Expressed as standard deviations. ** T-score −2.5 or below in the lumbar spine, femoral neck, total proximal femur, or 1/3 radius; low-trauma spine or hip fracture (regardless of bone mineral density); T-score between −1.0 and −2.5 and a fragility fracture of proximal humerus, pelvis, or distal forearm; T-score between −1.0 and −2.5 and high FRAX^®^ (fracture risk assessment tool) fracture probability based on country-specific threshold; *** trabecular bone score, expressed as low-intermediate if TBS < 1.31 g/cm^3^ and normal if TBS > 1.31 g/cm^3^; ^#^ as continuous value. TBS, trabecular bone score, expressed as g/cm^2^; SD, standard deviation; anterior (A)—10 mm anterior from the mental foramina, MF; molar (M)—10 mm posterior from the MF; posterior (P)—25 mm posterior from the MF; symphysis (S)—equidistant from the centers of the right and left MF.

## Data Availability

The raw data supporting the conclusions of this article will be made available by the authors on request.
